# Impact of death education programs on nurses’ and nursing students’ mortality perceptions and end-of-life coping competencies: a decade-long systematic review and meta-analysis

**DOI:** 10.3389/fmed.2026.1791470

**Published:** 2026-05-26

**Authors:** Fan Sun, Jing Tang, Weifeng Wang, Yuchen Wang, Xueling Qiu, Chenxi Sun, Wenjuan Cao, Lu Tang

**Affiliations:** 1 School of Nursing, Shandong University of Traditional Chinese Medicine, Jinan, China; 2 Intensive Care Unit, the 960th Hospital of the People’s Liberation Army of China (PLA), Jinan, China; 3 School of Nursing, Shandong Second Medical University, Weifang, China; 4 School of Nursing, Jinzhou Medical University, Jinzhou, China; 5 School of Nursing, Shandong First Medical University, Taian, China; 6 Department of Stomatology, the 960th Hospital of the People’s Liberation Army of China (PLA), Jinan, China

**Keywords:** attitude to death, death education, end-of-life care competence, meta-analysis, nurses, nursing students

## Abstract

**Objectives:**

To evaluate the effects of death education on nurses’ and nursing students’ attitudes toward death and end-of-life care competence.

**Design:**

Systematic review and meta-analysis of RCTs and CCTs.

**Data sources:**

Seven databases (2014–2025) including PubMed, Embase, CNKI, etc.

**Review methods:**

Two reviewers independently screened, extracted data, assessed risk of bias (RoB 2.0), and rated evidence certainty (GRADE). Random-effects meta-analyses, subgroup, and sensitivity analyses were performed.

**Results:**

Thirteen studies (*N* = 1,142) were included. Death education significantly improved end-of-life care competence (SMD = 1.05, 95% CI: 0.76–1.34, *p* < 0.001, I^2^ = 54%, moderate certainty). However, no significant effect was found on attitudes toward death (SMD = 0.25, 95% CI: −0.17 to 0.66, *p* = 0.24, I^2^ = 83%, very low certainty). Subgroup analyses showed consistent benefits for online and in-person training. Sensitivity analyses confirmed robustness. Most studies had high risk of bias, and only 3/13 included follow-up.

**Conclusion:**

Death education may enhance end-of-life care skills, but its impact on death attitudes is uncertain. Evidence is limited by high risk of bias, heterogeneity, and lack of long-term follow-up. Future rigorous RCTs with longer follow-up are required. Findings should be considered hypothesis-generating.

## 1 Introduction

### 1.1 Background

According to the World Health Organization (1), the number of deaths worldwide would reach 61–63 million in 2024, highlighting the urgency of hospice care. Nurses, as primary caregivers for dying patients, play a critical role in this context. Their attitudes toward death and their end-of-life care competence directly influence both patient outcomes and their own professional well-being (2). Negative death attitudes among nurses have been linked to higher burnout and turnover, whereas positive attitudes are associated with improved care quality and job satisfaction (3, 4, 5).

Death education aims to guide people to understand and treat death scientifically and humanely, to shift death attitudes and behaviors, and to appreciate the value and meaning of life, so that they can face death with a positive attitude (6). However, death education still faces challenges in nursing practice and education. Globally, death is often seen as a taboo subject, especially in Eastern cultures, where traditional beliefs limit its acceptance among nursing students and nurses (7). In recent years, death education programs have been implemented in many countries, but their efficacy lacks robust evidence. Some studies report significant improvements in death attitudes, while others show limited effects depending on cultural background and course length (6). Therefore, the effectiveness of death education, optimal delivery formats and durations, and sources of heterogeneity across Eastern and Western cultures need systematic investigation.

Previous reviews on death education have often been limited by narrow scope, inconsistent outcome definitions, or lack of cultural comparative analysis. To address these gaps, this review aims to systematically evaluate the effects of death education interventions on both death attitudes and end-of-life care competence among nurses and nursing students, and to explore how intervention characteristics and cultural context moderate these effects. Findings are intended to inform evidence-based curriculum development and policy recommendations in nursing education.

## 2 Methods

This review followed the PRISMA 2020 guidelines. The protocol was registered in PROSPERO (CRD42024607939).

### 2.1 Eligibility criteria

#### 2.1.1 Population

Nurses (any clinical setting) and nursing students (any year of study).

#### 2.1.2 Intervention

Structured death education programs, including but not limited to courses, workshops, simulations, or digital modules focusing on end-of-life care, grief support, or palliative care.

#### 2.1.3 Comparator

Included control studies with no intervention in the control group, placebo control (receiving educational intervention for non-end-of-life issues) or waitlist control.

#### 2.1.4 Outcomes

Primary: self-reported attitudes toward one’s own death (e.g., death anxiety, death acceptance, death avoidance), as measured by validated instruments such as the Death Attitude Profile-Revised (DAP-R). Secondary: self-reported or observed competence in end-of-life care.

### 2.2 Study design

The inclusion criteria encompassed randomized controlled trials (RCTs), cluster-based designs, and controlled clinical trials (CCTs). Given the limited number of published RCTs in this emerging field, we included controlled clinical trials (CCTs) to provide a more comprehensive evidence synthesis. Sensitivity analyses excluding CCTs confirmed the direction and significance of effects, indicating that their inclusion did not distort the main findings.

### 2.3 Language and publication status

Eligible materials encompassed peer-reviewed journal publications and unpublished manuscripts originally composed or translated into English or Chinese.

### 2.4 Study selection

A systematic literature search was performed across five English-language databases (PubMed, Embase, Web of Science, Ovid, and Cochrane) and two Chinese databases (CNKI and Wanfang Data) for studies published between January 1, 2014, and December 31, 2025. Search terms included “death education,” “end-of-life training,” “nurse,” “nursing student,” “attitude to death,” and “coping with death.” The full search strategy is provided in Supplementary File 1. Manual screening of reference lists from relevant reviews and selected articles was complemented by backward citation tracking to identify additional sources. All retrieved records were imported into EndNote 21 for organization by source and elimination of duplicates. Initial screening of titles and abstracts was performed against predefined eligibility criteria, followed by full-text evaluations of potentially eligible studies to assess relevance. Two independent reviewers executed the selection process, with discrepancies resolved through consensus-based discussions.

### 2.5 Data extraction

Two reviewers independently screened titles/abstracts and full texts using a piloted, custom data extraction form. Discrepancies were resolved through discussion. Extracted data included study characteristics, participant demographics, intervention details, outcome measures, and results. Quantitative outcomes were recorded as means and standard deviations, with missing values calculated through validated statistical transformations (8).

### 2.6 Risk of bias and quality of the evidence

Two independent investigators assessed risk of bias using the revised Cochrane RoB 2.0 tool to examine potential bias in the randomization process, allocation concealment, performance and detection bias, missing outcome data, outcome measures, and selection of results reported by all randomized trials (9). The quality of evidence was assessed using the GRADE (Grading of Recommendations Assessment, Development and Evaluation) approach. The GRADE approach assessed the quality of the evidence by considering study limitations, indirectness, inconsistency, imprecision, and publication bias (10).

### 2.7 Data synthesis

The characteristics of the included studies and interventions are summarized narratively. A meta-analysis was performed using Review Manager software 5.3 to pool data for the same outcomes in a random-effects model. All of the included studies reported results using continuous data and different scales, standardized mean differences (SMD) and 95% confidence intervals (CI) were used as effect measures under the inverse variance method (8). Heterogeneity was assessed by reporting 95% prediction intervals and I^2^ statistics, where possible. SMDs were interpreted as: 0.2 = small, 0.5 = medium, 0.8 = large effect. For the chi-square test, statistically significant heterogeneity was identified when its corresponding *p*-value was less than 0.10 (8). To reduce the level of heterogeneity and analyze the impact of certain variables on the results studied, subgroup analyses were performed (8, 11). The variables examined were ethnicity, duration of intervention, and method of intervention delivery. To assess the robustness of the meta-analysis findings, sensitivity analyses were planned *a priori*. We conducted two sets of sensitivity analyses for both primary outcomes (attitudes toward death and end-of-life care competence). By risk of bias: excluding studies judged to have a high overall risk of bias. By study design: excluding non-randomized controlled clinical trials (CCTs) to evaluate the impact of including studies with lower internal validity.

## 3 Results

### 3.1 Search results

**FIGURE 1:**
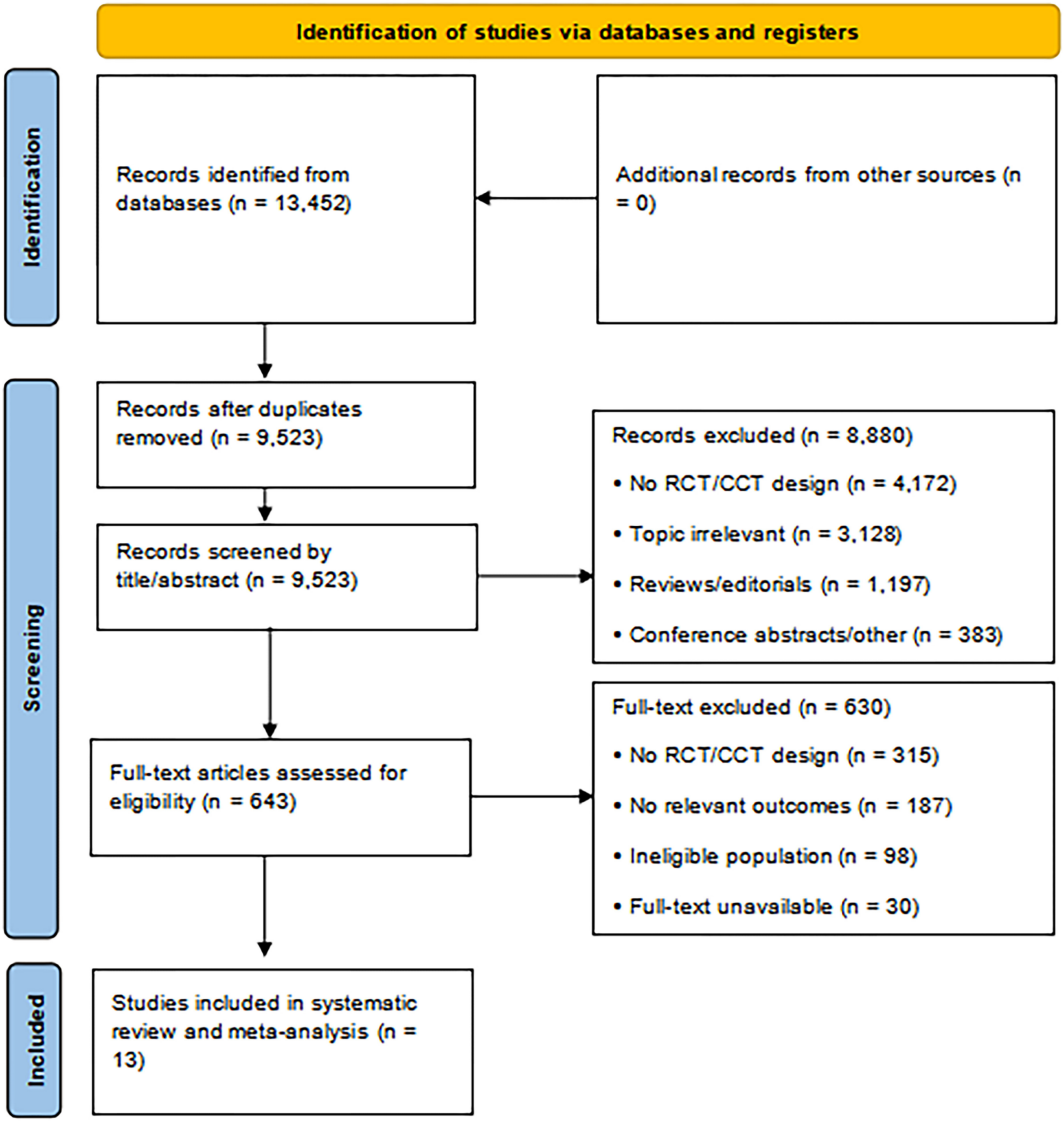
PRISMA 2020 flow diagram of study identification, screening, eligibility, and inclusion.

Database searches yielded 13,452 records. After deduplication and screening, 13 studies (8 RCTs, 5 CCTs) involving 1,142 participants were included (Figure 1). The 13 articles (7, 12–23), all peer-reviewed original studies, were included in the review. The PRISMA flowchart summarizing the search and selection process is shown in Figure 1.

### 3.2 Characteristics of the included studies

The systematic review incorporated 13 multinational studies spanning seven countries: China (*n* = 3), Iran (*n* = 2), Japan (*n* = 1), South Korea (*n* = 2), Turkey (*n* = 2), Spain (*n* = 1), and the United States (*n* = 2). These comprised 5 controlled clinical trials (CCTs) and 8 randomized controlled trials (RCTs) (7, 13–15, 17, 19, 22, 23). Eight investigations focused on nurses across diverse clinical departments, including emergency care (21), oncology (7), and intensive care units (13), while five examined nursing students. Educational interventions utilized in-person workshops (*n* = 9) and online (*n* = 4) approaches: in-person workshops employed multimodal strategies such as case-based role-playing, group discussions, and video-assisted learning to deliver hospice care training. In contrast, online modules leveraged digital platforms for asynchronous lectures, curated literature reviews, and structured peer discussions, supplemented by video-based instruction (12, 20, 21). A summary of participant characteristics can be found in Supplementary Table 1.

### 3.3 Quality appraisal

**FIGURE 2:**
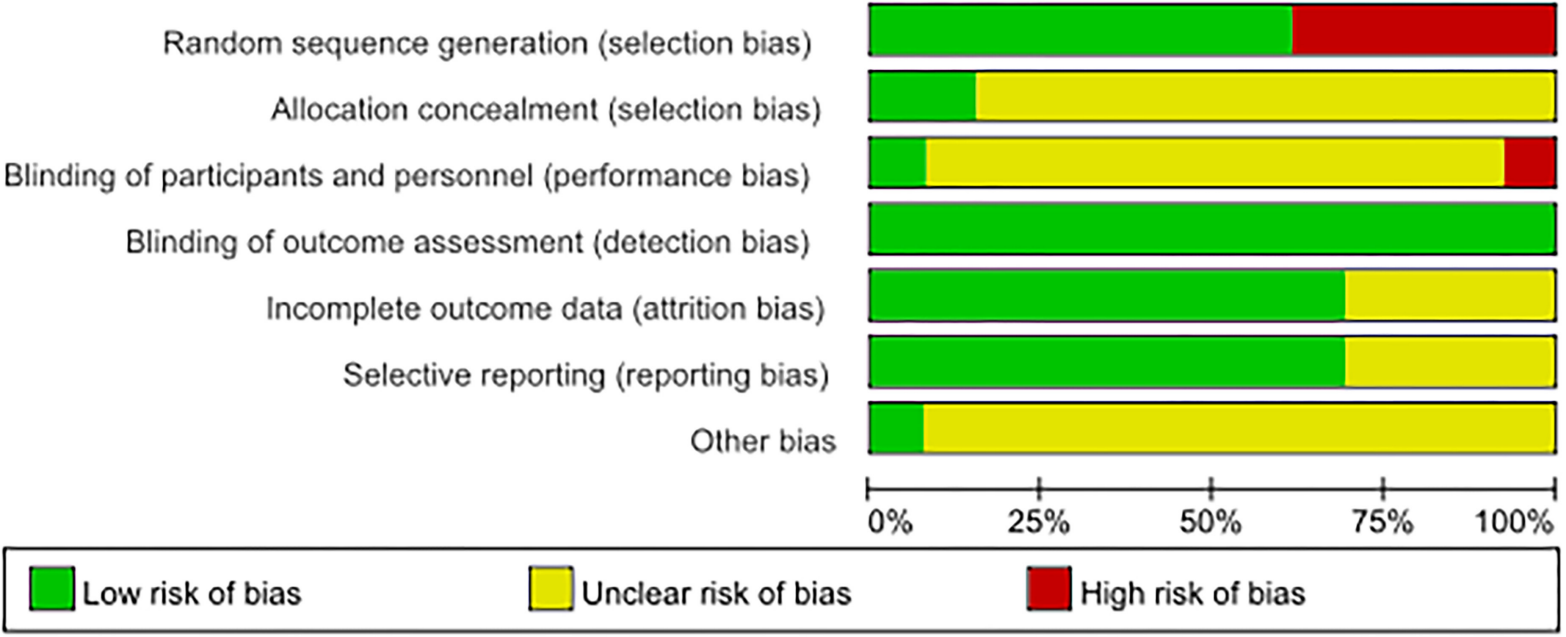
Risk of bias graph: summary of risk of bias items across all included studies.

The methodological rigor of included studies was evaluated using the Cochrane Risk of Bias tool, with the majority of studies classified as having high or unclear risk of bias, primarily due to lack of blinding and inadequate randomization. Inter-rater agreement was high (κ = 0.97). A visual representation of bias distribution across domains is illustrated in Figure 2. Evidence certainty was appraised using the GRADE framework, revealing “very low” confidence in outcomes related to death attitudes and “moderate” confidence in those addressing coping with death. A comprehensive GRADE evidence profile is provided in the Supplementary File 2, Supplementary Table 1.

### 3.4 Attitude toward death

#### 3.4.1 Primary meta-analysis of death attitudes

**FIGURE 3:**
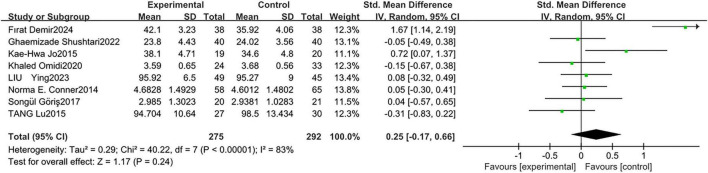
Forest plot of the effect of death education on attitudes toward death at post-intervention (eight studies).

The pooled analysis of eight studies evaluating immediate post-intervention death attitudes (7, 12, 13, 15–17, 21, 22) revealed a non-significant overall effect (SMD = 0.25, 95% CI: −0.17 to 0.66, *Z* = 1.17, *p* = 0.24), with substantial heterogeneity (I^2^ = 83%) (Figure 3). Subgroup analyses by duration, delivery mode, and region did not explain heterogeneity

#### 3.4.2 Subgroup analyses

Subgroup analyses of the Asian and Caucasian lineages showed that no significant differences were observed in the subgroups of the Asian lineages (SMD = 0.13, 95% CI: −0.38 to 0.64, *p* = 0.61, I^2^ = 66%) nor in the Caucasian lineages (SMD = 0.31, 95% CI: −0.31 to 0.93, *p* = 0.33, I^2^ = 88%) (Supplementary File 3, Supplementary Figure 1a).

Subgroup analyses of the length of the intervention showed that no significant differences were observed in the subgroups up to 1 month (SMD = 0.38, 95% CI: −0.47 to 1.22, *p* = 0.39, I^2^ = 91%), nor in the subgroups longer than 1 month (SMD = 0.09, 95% CI: −0.23 to 0.42, *p* = 0.57, I^2^ = 49%) (Supplementary File 3, Supplementary Figure 1b).

Subgroup analyses of the intervention modalities showed that no significant differences were observed in the subgroup trained in the online modality (SMD = 0.58, 95% CI: −0.33 to 1.50, *p* = 0.21, I^2^ = 93%), nor in the subgroup trained in the in-person workshops modality (SMD = 0.01, 95% CI: −0.30 to 0.32, *p* = 0.94, I^2^ = 38%) (Supplementary File 3, Supplementary Figure 1c).

Subgroup analyses for nurses and nursing students revealed no significant differences in the nursing student subgroup (SMD = 0.33, 95% CI: −0.31 to 0.97, *p* = 0.31, I^2^ = 68%), nor in the nurse subgroup (SMD = 0.21, 95% CI: −0.34 to 0.76, *p* = 0.45, I^2^ = 87%) (Supplementary File 3, Supplementary Figure 1d).

### 3.5 Capacity for coping with death

#### 3.5.1 Primary meta-analysis of end-of-life care outcomes

**FIGURE 4:**
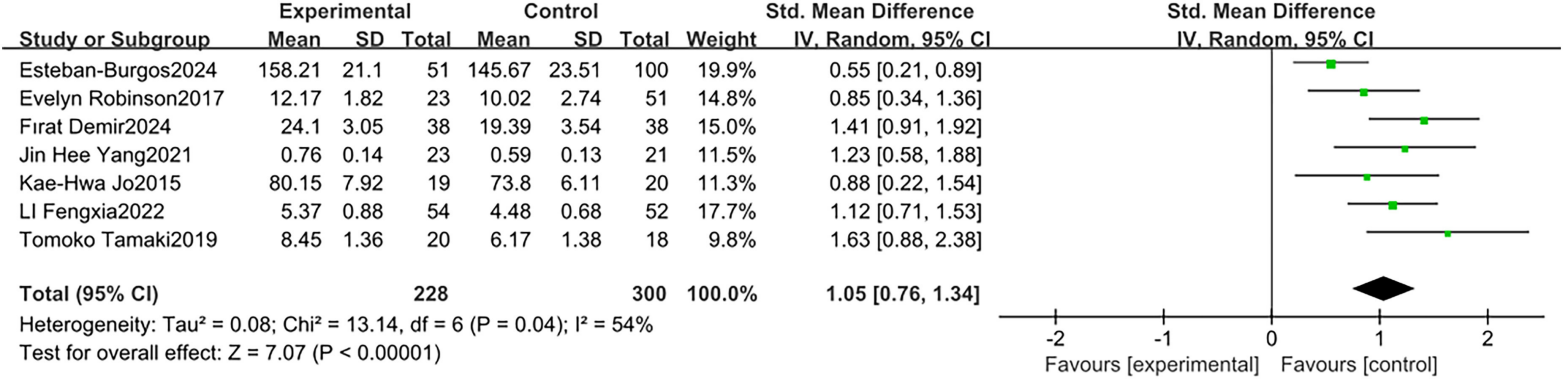
Forest plot of the effect of death education on end-of-life care competence at post-intervention (seven studies).

The synthesis of seven studies evaluating immediate post-intervention outcomes in end-of-life care (13, 14, 16, 18–20, 23) demonstrated a statistically robust overall effect (SMD = 1.05, 95% CI: 0.76–1.34, *Z* = 7.07, *p* < 0.00001), with moderate heterogeneity (I^2^ = 54%) (Figure 4). Subgroup analyses suggested stronger and more consistent effects in online interventions and Asian subgroups.

#### 3.5.2 Subgroup analyses

Subgroup analysis of Asian and Caucasian lineages showed that significant differences were observed in the Asian lineage subgroup (SMD = 1.17, 95% CI: 0.89–1.46, *p* < 0.00001, I^2^ = 0%). Significant differences were also observed in the Caucasian lineage (SMD = 0.91, 95% CI: 0.40–1.42, *p* < 0.00001, I^2^ = 74%). However, the effect was stronger and the consistency of results was higher for subgroups of sublineages (Supplementary File 3, Supplementary Figure 2a).

Subgroup analyses of the length of the intervention showed that significant differences were observed in subgroups of up to 1 month (SMD = 1.04, 95% CI: 0.63–1.46, *p* < 0.00001, I^2^ = 70%), and in subgroups of more than 1 month (SMD = 1.07, 95% CI: 0.61–1.52, *p* < 0.00001, I^2^ = 36%). Heterogeneity was higher for short-term interventions and lower for long-term interventions, with more stable outcomes (Supplementary File 3, Supplementary Figure 2b).

Subgroup analyses of the intervention modalities showed that significant differences were observed in the subgroup trained in the online modality (SMD = 1.34, 95% CI: 0.95–1.74, *p* < 0.00001, I^2^ = 0%), as well as in the subgroup trained in the in-person workshops modality (SMD = 0.94, 95% CI: 0.61–1.28, *p* < 0.00001, I^2^ = 55%). Both online and in-person workshops training produced significant intervention effects, but online training had greater effect strength and greater consistency of results (Supplementary File 3, Supplementary Figure 2c).

Analysis of the subgroups of nurses and nursing students revealed a significant difference in the nursing student subgroup (SMD = 0.99, 95% CI: 0.53–1.44, *p* < 0.0001, I^2^ = 65%). A significant difference was also observed in the nurse subgroup (SMD = 1.24, 95% CI: 0.95–1.52, *p* < 0.00001, I^2^ = 0%). However, the effect was stronger and the consistency of results was higher for the nurses subgroup (Supplementary File 3, Supplementary Figure 2d).

Analysis of the simulation and lecture subgroups revealed a significant difference in the simulation subgroup (SMD = 1.03, 95% CI: 0.46–1.60, *p* < 0.0004, I^2^ = 77%). A significant difference was also observed in the lecture subgroup (SMD = 1.11, 95% CI: 0.82–1.39, *p* < 0.00001, I^2^ = 0%). However, the lecture subgroup demonstrated stronger effects and greater consistency in results (Supplementary File 3, Supplementary Figure 2e).

### 3.6 Sensitivity analyses

#### 3.6.1 Sensitivity analysis for attitudes toward death

Excluding high risk of bias studies: after removing three studies judged to have a high overall risk of bias (12, 21), the pooled effect size from the remaining five studies remained non-significant (SMD = 0.24, 95% CI: −0.47 to 0.94, *p* = 0.51). Heterogeneity remained substantial (I^2^ = 89%) (Supplementary File 4, Supplementary Figure 1a).

Excluding non-randomized studies (CCTs): after excluding the two CCTs (12, 21) from the analysis of eight studies, the pooled effect from the six RCTs was still non-significant (SMD = 0.31, 95% CI: −0.30 to 0.93, *p* = 0.31), with considerable heterogeneity (I^2^ = 87%) (Supplementary File 4, Supplementary Figure 1b).

#### 3.6.2 Sensitivity analysis for end-of-life care competence

Excluding high risk of bias studies: removing two studies with a high overall risk of bias (18, 20) from the seven studies resulted in a slightly increased but still large and significant effect size (SMD = 1.07, 95% CI: 0.68–1.47, *p* < 0.00001). Heterogeneity decreased to a moderate level (I^2^ = 68%) (Supplementary File 4, Supplementary Figure 2a).

Excluding non-randomized studies (CCTs): excluding the three CCTs (16, 18, 20) yielded a pooled effect from four RCTs of SMD = 1.12, 95% CI: 0.64–1.60, *p* < 0.00001, with moderate heterogeneity (I^2^ = 75%) (Supplementary File 4, Supplementary Figure 2b).

Interpretation: the sensitivity analyses confirmed the robustness of the main findings. The non-significant effect on death attitudes and the significant, large effect on care competence remained unchanged regardless of the exclusion criteria. The reduction in heterogeneity for the competence outcome after excluding high-risk studies suggests that methodological quality contributes to the variability in observed effects for this outcome (Supplementary File 4).

### 3.7 Publication bias

Publication bias was assessed using funnel plots for the two primary outcomes (Supplementary File 3, Supplementary Figure 3 for end-of-life care competence; Supplementary File 3, Supplementary Figure 4 for attitudes toward death). For end-of-life care competence, all studies were symmetrically distributed around the pooled effect estimate, indicating no clear evidence of publication bias. For attitudes toward death, the funnel plot showed slight asymmetry with one study located outside the pseudo-confidence limits. This asymmetry is likely attributable to the substantial clinical and methodological heterogeneity among the included studies (I^2^ = 83%) rather than to publication bias *per se*. Due to the limited number of studies (k < 10 for both outcomes), formal statistical tests such as Egger’s regression test were not performed because they have low power to detect asymmetry when the number of studies is small.

## 4 Discussion

This study, for the first time through a systematic review and meta-analysis, evaluated the effects of death education on nurses’ and nursing students’ attitudes toward death and end-of-life care competency. The results indicate that death education can significantly and consistently improve end-of-life care competence, but its short-term effect on attitudes toward death is limited and highly heterogeneous. This differentiation reveals the fundamental differences in the mechanisms and intervention pathways between “attitude change” and “skill acquisition,” providing key evidence for the design of future targeted educational strategies.

### 4.1 Explanation and integration of key findings

Our meta-analysis indicates that the effect size for improving end-of-life care competencies is large (SMD = 1.05), with moderate-quality evidence, suggesting that death education has a clear and positive impact on skills training. This aligns with theoretical models of skill acquisition–structured, repeatable clinical skills [such as the SPIKES communication model (24), pain assessment, and grief counseling] can be effectively taught and internalized through demonstration, practice, and immediate feedback, with online standardized courses showing significant advantages in this process (19, 20).

In contrast, attitudes toward death did not show consistent positive changes (SMD = 0.25, very low-quality evidence). This finding contradicts some prior studies (25) but highlights the complexity of attitudes as deep psychological constructs. Attitudes are rooted in an individual’s cultural background, religious beliefs, personal experiences, and emotional encounters (26, 27; 28), and short-term, standardized educational interventions are unlikely to shift these long-established cognitive and emotional frameworks. The high heterogeneity observed in this study (I^2^ = 83%) further supports this view, indicating considerable variability in intervention effects across different cultural or personal backgrounds.

### 4.2 Analysis of the key reasons for limited changes in attitudes toward death

#### 4.2.1 Cultural taboos and measurement bias

In many cultures, especially East Asian cultures, death remains a social taboo (29). Participants may hide their true death anxiety or negative attitudes due to social desirability bias in scale assessments, resulting in measurement outcomes that do not reflect actual changes. This cultural constraint requires future interventions and evaluations to adopt more culturally sensitive qualitative or mixed methods (30).

#### 4.2.2 Mismatch between intervention content and depth

Most included studies employed short-term interventions (ranging from a few hours to a few weeks) primarily focused on knowledge dissemination. However, deep attitude changes require engagement with emotions and values, which may depend on long-term, experiential, and reflective learning processes (e.g., “death cafés,” narrative therapy, clinical reflection journals), and these are insufficiently applied in current interventions (31).

#### 4.2.3 Conceptual confusion in assessment tools

Different studies used instruments measuring potentially distinct subdimensions of attitudes toward one’s own death (e.g., DAP-R, short-form DAP-R, locally developed scales). This conceptual variation likely contributes to the high heterogeneity. Future research needs to clearly define and distinguish between “attitudes toward one’s own death” and “attitudes toward providing end-of-life care.” The subgroup analysis for attitudes by participant type included only two studies in nursing students; therefore, the null finding should be interpreted with caution and requires confirmation in future studies.

### 4.3 Key factors for improving end-of-life care competence

#### 4.3.1 The “technological neutrality” and transferability of skills training

Whether delivered online or offline, evidence-based standardized nursing skills (such as symptom management and communication procedures) can be effectively taught. Online formats, in particular, demonstrate advantages of large effect sizes and low heterogeneity, suggesting that through modular design, scenario simulation, and instant testing, efficient and consistent skills training can be achieved (13).

#### 4.3.2 Integration of simulation and practice

Projects with significant effects in the included studies all integrated high-fidelity simulations, role-playing, or clinical practice (19). This “learning by doing” approach directly translates knowledge into behavior and reinforces learning outcomes through feedback, making it an effective method for competency development.

#### 4.3.3 Insights from subgroup analysis

The Asian subgroup showed higher consistency and homogeneity in competency improvement, which may be due to the fact that studies in this subgroup more frequently used structured, clearly objective standardized skills courses. This suggests that clearly structured and culturally adapted course designs may better predict intervention outcomes than a general cultural affiliation.

### 4.4 Sources of heterogeneity and study limitations

This review has several methodological limitations that warrant careful consideration, as highlighted by the reviewer.

Risk of bias related to randomization and blinding. The majority of included studies (8 out of 13) had a high or unclear risk of bias, primarily due to inadequate randomization procedures (e.g., lack of allocation concealment, absence of blinding of participants and personnel). As noted in the quality appraisal (Section “3.3 Quality appraisal”), most studies were rated as having high or unclear risk of bias. This is a substantial limitation because unblinded designs in educational interventions may inflate effect estimates, particularly for self-reported outcomes such as attitudes toward death. Consequently, our pooled effect for death attitudes (SMD = 0.25) may be overestimated, and the true effect could be even closer to zero. The sensitivity analysis excluding high-risk studies confirmed the non-significant finding, indicating that the result is robust to bias, but the low certainty of evidence remains.

GRADE evidence certainty and its implications. The GRADE assessment revealed very low certainty for the attitude-toward-death outcome and moderate certainty for end-of-life care competence. The very low rating for death attitudes was primarily due to: (a) serious risk of bias across most studies; (b) substantial inconsistency (I^2^ = 83%); (c) imprecision due to small sample sizes; and (d) indirectness because many studies measured attitudes using different scales. A critical contributor to the downgrading was the lack of follow-up assessments–only 3 out of 13 studies included any post-intervention follow-up beyond immediate measurement. Without follow-up, we cannot determine whether any observed changes in attitudes or competence are sustained over time. This limitation is particularly important for attitude change, which, if it occurs, would be expected to require longer-term reinforcement. Therefore, our conclusions regarding the short-term impact of death education on attitudes should be interpreted with caution, and the absence of follow-up precludes any inference about long-term effectiveness.

Appropriateness of conducting a meta-analysis given these limitations. The reviewer raised a concern about whether it was appropriate to pool studies with very low certainty evidence and high risk of bias. According to Cochrane guidelines, meta-analysis can be performed even when evidence quality is low, provided that the limitations are transparently reported and the pooled estimates are interpreted with appropriate caution. The purpose of this meta-analysis was not to claim definitive causal effects but to synthesize existing data to identify patterns and generate hypotheses. The non-significant pooled effect, despite substantial heterogeneity, itself provides an important message: death education as currently designed does not reliably change attitudes in the short term. The sensitivity analyses (excluding high-risk studies and non-randomized designs) confirmed the robustness of this finding. Thus, while the evidence is very low certainty, the meta-analysis serves a useful exploratory function and highlights the urgent need for better-designed trials with longer follow-up.

Additional limitations. Only Chinese and English literature were included, which may lead to regional publication bias. Most studies lacked long-term follow-up, as noted. Outcome indicators were primarily self-reported, with a lack of patient outcome data or objective behavioral observations. Randomized controlled trials (RCTs) and non-randomized controlled trials (CCTs) were combined in the analysis; despite sensitivity analyses showing consistent results, this may still affect the precision of the estimated effects. Finally, key moderators such as individual levels of occupational burnout, frequency of exposure to death, and religious beliefs were not systematically measured or reported across studies (32, 33; 34). Further sources of heterogeneity include intervention type (simulation versus lecture), outcome measurement tools, and participant type (nurses versus nursing students). Meta-regression could be used to explore these factors in future reviews with larger sample sizes.

### 4.5 Implications for future research and practice

Based on the differentiated characteristics of the “attitude-ability” effect, this study proposes a “dual-track” strategy for death education: the ability enhancement track focuses on promoting standardized skill courses that are online and modular, drawing on international palliative care education frameworks such as EPEC. It involves developing digital micro-courses covering core skills and incorporating them into mandatory continuing education for nurses in high-death-exposure departments such as oncology and ICU. The attitude transformation track emphasizes designing long-term, experiential, and culturally immersive reflective programs. Rather than seeking short-term “correction” of attitudes, it aims to help individuals construct personalized meanings of life and death by creating safe and open spaces for exchange, leveraging narrative sharing, artistic expression, cross-cultural dialogue, and other methods. Educators should work with humanities scholars, religious studies experts, and community leaders to jointly develop localized educational content. At the research and practical policy level, future efforts should employ mixed research methods to deeply explore the underlying mechanisms of attitude change, develop multidimensional assessment toolkits, integrate death education into core undergraduate nursing curricula and post-graduate training, and establish inter-institutional teaching resource libraries and faculty training networks. Crucially, future RCTs must include follow-up assessments of at least 6 months to evaluate the sustainability of any changes, and they should adhere to CONSORT guidelines to minimize risk of bias.

## 5 Conclusion

In summary, death education can effectively serve as a “skill accelerator” to enhance professional end-of-life care abilities, but its role as a “conceptual catalyst” for transforming deep-seated attitudes toward death is limited. The very low certainty of evidence, high risk of bias, and lack of follow-up in the included studies mean that these findings should be interpreted as hypothesis-generating rather than definitive. Future education and research should no longer treat “attitude change” as a simple outcome of knowledge transmission; instead, it should be understood as a complex personal growth journey that requires long-term respect, dialogue, and individualized support. Combining standardized skills training with human-centered attitude cultivation is the key approach to developing end-of-life caregivers who are both competent and compassionate.

## Supplementary material

Supplementary Figure 1 **(a)** Subgroup analysis of attitudes toward death by ethnicity (Asian vs. Caucasian). **(b)** Subgroup analysis of attitudes toward death by intervention duration (≤1 month vs. >1 month). **(c)** Subgroup analysis of attitudes toward death by delivery mode (online vs. in-person). **(d)** Subgroup analysis of attitudes toward death by participant type (nurses vs. nursing students).

Supplementary Figure 2 **(a)** Subgroup analysis of end-of-life care competence by ethnicity (Asian vs. Caucasian). **(b)** Subgroup analysis of end-of-life care competence by intervention duration (≤1 month vs. >1 month). **(c)** Subgroup analysis of end-of-life care competence by delivery mode (online vs. in-person). **(d)** Subgroup analysis of end-of-life care competence by participant type (nurses vs. nursing students). **(e)** Subgroup analysis of end-of-life care competence by teaching method (simulation vs. lecture). Publication bias funnel plots.

Supplementary Figure 3 Funnel plot for end-of-life care competence (publication bias assessment).

Supplementary Figure 4 Funnel plot for attitudes toward death (publication bias assessment).
