# Supplementary material for: Impact of death education programs on nurses’ and nursing students’ mortality perceptions and end-of-life coping competencies: a decade-long systematic review and meta-analysis
Source: Front Med (Lausanne). 2026 May 26;13:1791470. doi: 10.3389/fmed.2026.1791470 (PMC13246359; doi:10.3389/fmed.2026.1791470)
Supplement: Supplementary file 1 [file Table_1.docx]

Table 1 Summary of included studies.

| **Study (country)** | **Research design** | **Participants number** | **Age mean (SD) or mean (range) or range** | **Gender**  **(M/F)** | **Intervention** | **Control** | **Intervention duration**  **(follow-up)** | **Review outcomes: 1. Attitude toward death 2. Capacity for coping with death** |
| --- | --- | --- | --- | --- | --- | --- | --- | --- |
| ( LI Fengxia et al., 2022)PRC | RCT | Nurse of 106 Cancer Hospital | I.25.74±1.42 C.25.99±1.69 | I.M3/F51  C.M3/F49 | A self-constructed death education program | Hospital-level training | 9h four stages(4 weeks) | 1.no 2.yes |
| (Tanglu et al., 2015)PRC | RCT | 57 Emergency Nurses | I.<25:10,25~29:10,≥30:7 C.<25:16,25~29:9,≥30:5 | I.M2/F25  C.M2/F28 | Continuing Education Death Education Lecture Program | None | 11 lessons, 21 hours in total,1 class hour = 40 minutes(None) | 1.yes 2.no |
| (Liuying et al., 2023)PRC | CCT | 94 Emergency Nurses | I.31.02±5.27 C.29.00±4.79 | I.M4/F45  C.M4/F41 | "Qianchat Live". | Routine department business training | 8 months, 24 hours(None) | 1.yes 2.no |
| (Göriş et al., 2017)Turkey | RCT | 41 Cancer Hospital Nurse | none | none | palliative care courses | None | 90-min sessions of training a period of 3 weeks（3 month） | 1.yes 2.no |
| (Conner et al., 2014)US | CCT | 123 nursing  students | 18 to 36+ years old | I.M12/F46  C.M5/F60 | Online courses on end-of-life content | Online courses with no end-of-life content | 16-week（None） | 1.yes 2.no |
| (Esteban-Burgos et al., 2024)Spain | RCT | 264 nursing  students | mean age was  20.94 years old | M55/F219 | palliative care 150 h | None | 3-week(None) | 1.no 2.yes |
| (Demir et al., 2024)Turkey | RCT | 76 intensive care nurses | I.29.00±3.35 C.30.31±3.64 | M52/F24 | EoLC awareness education  programme on an online platform | None | 40–60-min sessions once a week for 4 weeks（None） | 1.yes 2.yes |
| (Yang & Shin, 2021)Korea | CCT | 44 intensive  care nurses | I.27.14±2.52 C.25.14±2.27 | M10/F34 | End-of-Life Care Mobile App | End-of-Life Care booklet | 30min×7days(None) | 1.no 2.yes |
| (Jo & An, 2015)Korea | CCT | 39 nursing  students | I.22.16±0.83 C.22.30±0.86 | M1/F38 | end-of-life care course | end-of-life care course | 2h×16weeks(None) | 1.yes 2.yes |
| (Robinson & Epps, 2017)US | CCT | 74nursing  students | 21 to 46 years | M6/F68 | palliative care course | None | every other week  for 2 hours(None) | 1.no 2.yes |
| (Omidi et al., 2020)Iran | RCT | 57nurses in  an oncology ward | I.28.17±5.95 C.27.55±4.89 | I.M0/F24  C.M1/F32 | workshop training | None | three sessions(3month) | 1.yes 2.yes |
| (Ghaemizade Shushtari et al., 2022)Iran | RCT | 80nurses in  an oncology ward | 33.00±4.13 | M5/F75 | ELNEC core course | None | 60~90min×5(None) | 1.yes 2.yes |
| (Tamaki et al., 2019)  Japan | RCT | 38nursing  students | I.21.3±0.62 C.21.0±0 | M1/F37 | EOL care simulation program | None | 80min(None) | 1.no 2.yes |
