# Supplementary material for: Impact of death education programs on nurses’ and nursing students’ mortality perceptions and end-of-life coping competencies: a decade-long systematic review and meta-analysis
Source: Front Med (Lausanne). 2026 May 26;13:1791470. doi: 10.3389/fmed.2026.1791470 (PMC13246359; doi:10.3389/fmed.2026.1791470)
Supplement: Supplementary file 2 [file Table_2.docx]

**Complete Search Strategies**

The following search strategies were used to identify relevant studies. Searches were conducted from January 1, 2014, to December 31, 2025. All searches were performed on December 31, 2025.

Search Strategy Table

| Database | Search Date | Search Strategy | Records Retrieved |
| --- | --- | --- | --- |
| PubMed | Dec 31, 2025 | ("Death Education"[Mesh] OR "death education"[tiab] OR "end-of-life education"[tiab] OR "palliative care education"[tiab] OR "hospice education"[tiab]) AND ("Nurses"[Mesh] OR "Nursing Students"[Mesh] OR nurse*[tiab] OR "nursing student*"[tiab]) AND ("Attitude to Death"[Mesh] OR "death attitude*"[tiab] OR "death anxiety"[tiab] OR "coping with death"[tiab] OR "end-of-life care competency"[tiab]) AND ("Randomized Controlled Trial"[pt] OR "Controlled Clinical Trial"[pt] OR "clinical trial"[tiab] OR random*[tiab]) AND ("2014/01/01"[Date - Publication] : "2025/12/31"[Date - Publication]) | 2,845 |
| Embase | Dec 31, 2025 | ('death education'/exp OR 'death education':ti,ab OR 'end-of-life education':ti,ab OR 'palliative care education':ti,ab) AND ('nurse'/exp OR 'nursing student'/exp OR nurse*:ti,ab OR 'nursing student*':ti,ab) AND ('attitude to death'/exp OR 'death attitude*':ti,ab OR 'death anxiety':ti,ab OR 'coping with death':ti,ab) AND ('randomized controlled trial'/exp OR 'controlled clinical trial'/exp OR random*:ti,ab) AND [2014-2025]/py | 3,122 |
| Web of Science | Dec 31, 2025 | TS=("death education" OR "end-of-life education" OR "palliative care education") AND TS=(nurse* OR "nursing student*") AND TS=("death attitude*" OR "death anxiety" OR "coping with death") AND TS=(random* OR trial OR "controlled study") AND PY=(2014-2025) | 2,901 |
| Cochrane Library | Dec 31, 2025 | ("death education" OR "end-of-life education" OR "palliative care education") AND (nurse* OR "nursing student*") AND ("death attitude*" OR "death anxiety" OR "coping with death") in Title, Abstract, Keywords AND in Trials | 887 |
| Ovid MEDLINE | Dec 31, 2025 | 1. exp Death Education/ 2. (death education or end-of-life education or palliative care education).ti,ab. 3. 1 or 2 4. exp Nurses/ or exp Students, Nursing/ 5. (nurse* or nursing student*).ti,ab. 6. 4 or 5 7. exp Attitude to Death/ 8. (death attitude* or death anxiety or coping with death).ti,ab. 9. 7 or 8 10. exp Randomized Controlled Trials/ or exp Controlled Clinical Trials/ 11. (random* or trial).ti,ab. 12. 10 or 11 13. 3 and 6 and 9 and 12 14. limit 13 to yr="2014-2025" | 2,765 |
| CNKI | Dec 31, 2025 | SU=('死亡教育' + '临终教育' + '安宁疗护教育') AND SU=('护士' + '护生' + '护理学生') AND SU=('死亡态度' + '死亡焦虑' + '临终护理能力') AND FT=('随机' + '对照' + '实验') AND YE=('2014'–'2025') | 423 |
| Wanfang Data | Dec 31, 2025 | 主题:("死亡教育" OR "临终教育") AND 主题:("护士" OR "护生") AND 主题:("死亡态度" OR "临终护理") AND 文献类型:("随机对照试验" OR "临床对照试验") AND 年份:2014-2025 | 509 |
| Total before deduplication |  |  | 13,452 |

Notes on Search Strategy

1. Search Adaptation: The strategy was conceptually consistent across databases but adapted to each platform's specific syntax and controlled vocabulary (e.g., MeSH in PubMed, Emtree in Embase).
2. Language: No language restrictions were applied during the search, though only studies published in English or Chinese were included in the final synthesis.
3. Grey Literature: Conference abstracts, dissertations, and trial registries ([ClinicalTrials.gov](https://clinicaltrials.gov/), WHO ICTRP) were also scanned, though no additional eligible studies were identified beyond the database searches.
4. Manual Searching: Reference lists of all included studies and relevant systematic reviews were hand-searched, but no new eligible studies were found through this method.
5. Search Updates: The search was updated at the time of manuscript submission (December 2025) to ensure inclusion of the most recent evidence.
