# Supplementary material for: Impact of death education programs on nurses’ and nursing students’ mortality perceptions and end-of-life coping competencies: a decade-long systematic review and meta-analysis
Source: Front Med (Lausanne). 2026 May 26;13:1791470. doi: 10.3389/fmed.2026.1791470 (PMC13246359; doi:10.3389/fmed.2026.1791470)
Supplement: Supplementary file 3 [file Table_3.docx]

GRADE summary of evidence

| **Outcome** | **No. of studies** | **Study design** | **Risk of bias** | **Inconsistency** | **Indirectness** | **Imprecision** | **Publication bias** | **Effect size (95% CI)** | **Certainty** | **Importance** |
| --- | --- | --- | --- | --- | --- | --- | --- | --- | --- | --- |
| ****Death Attitude**** | 8 | RCT | Serious * | Not Serious | Not Serious | Serious c | Undetected | SMD 0.25 (-0.17, 0.66) | ****Low**** | Important |
| ****Coping Ability**** | 7 | RCT | Not Serious | Serious b | Not Serious | Not Serious | Undetected | SMD 0.98 (0.79, 1.16) | ****Moderate**** | Critical |
