# Supplementary material for: Impact of death education programs on nurses’ and nursing students’ mortality perceptions and end-of-life coping competencies: a decade-long systematic review and meta-analysis
Source: Front Med (Lausanne). 2026 May 26;13:1791470. doi: 10.3389/fmed.2026.1791470 (PMC13246359; doi:10.3389/fmed.2026.1791470)

Figure S1a Forest plot of sensitivity analysis for death attitudes (excluding studies with high risk of bias)


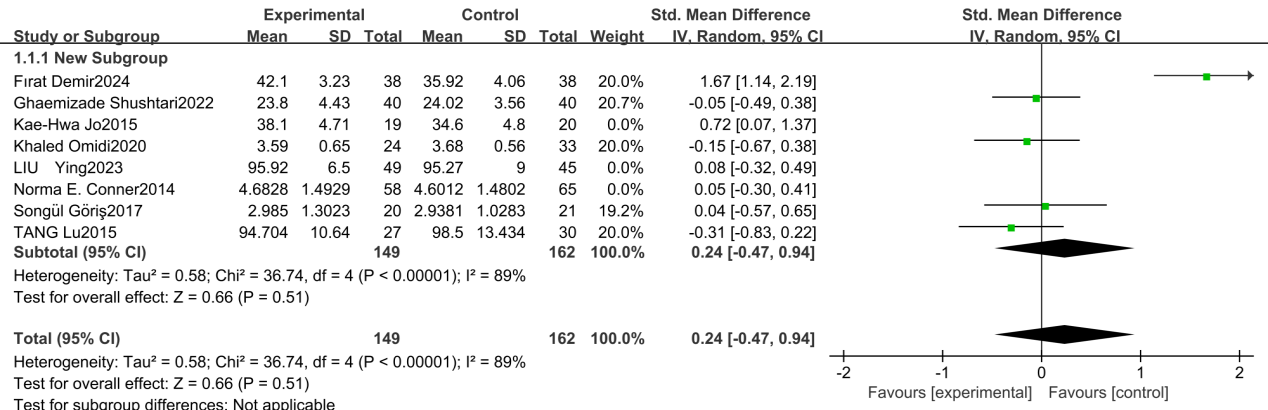


Figure S1b Forest plot of sensitivity analysis for death attitudes (excluding non‑randomized studies, CCTs)


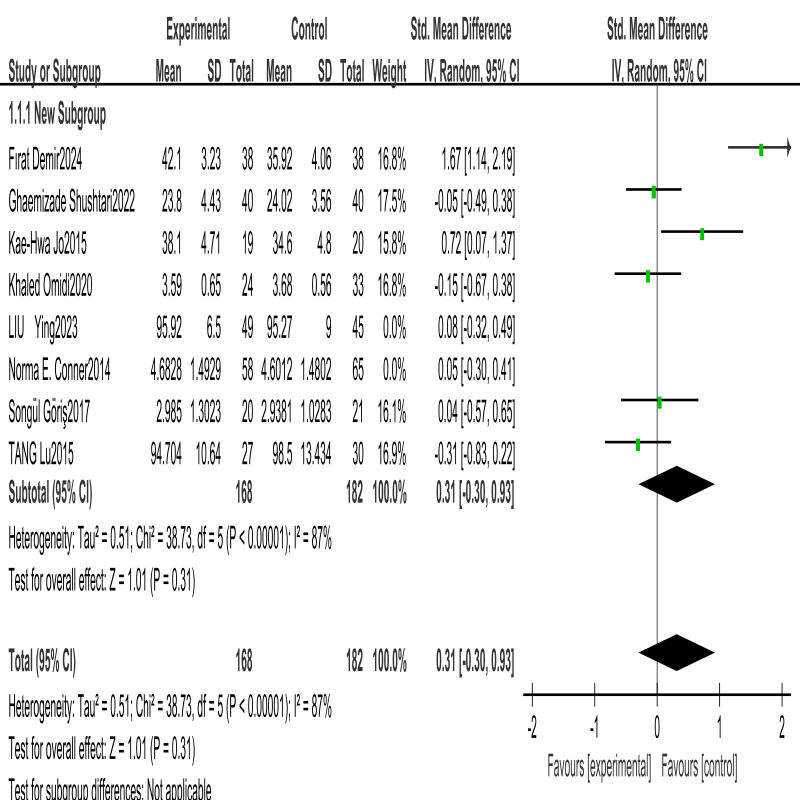


Figure S2a Forest plot of sensitivity analysis for end‑of‑life care competence (excluding studies with high risk of bias)


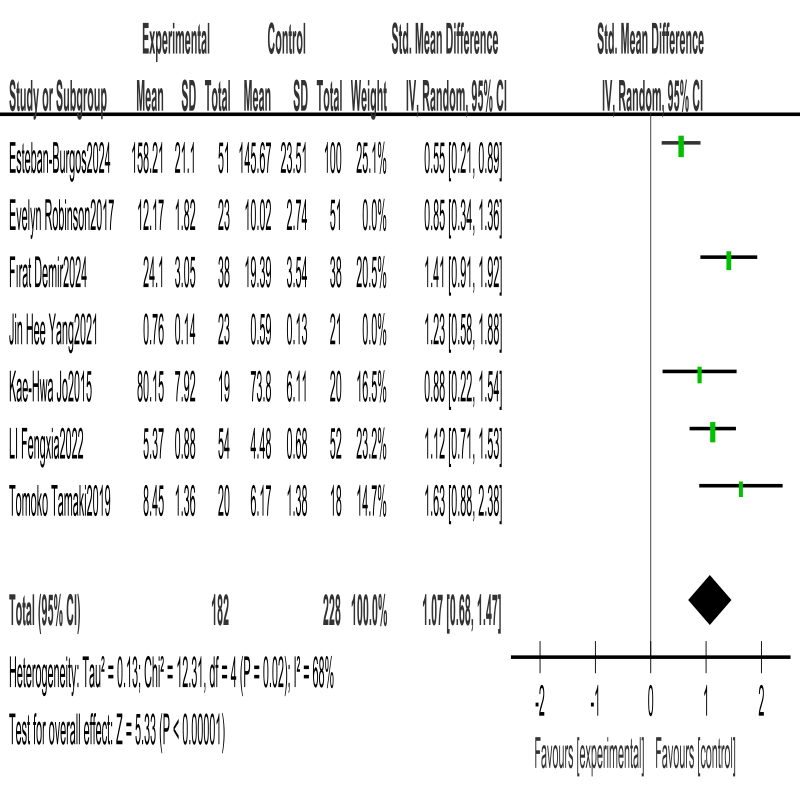


Figure S2b Forest plot of sensitivity analysis for end‑of‑life care competence (excluding non‑randomized studies, CCTs)


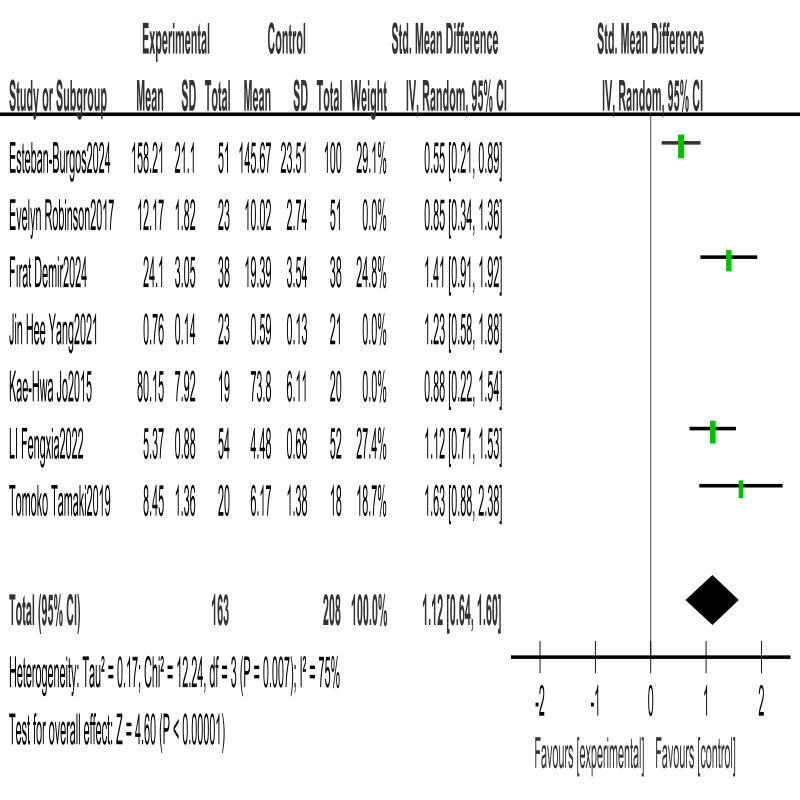

Supplement: Supplementary file 4 [file Table_5.docx]
