# Supplementary figures and images for: Impact of death education programs on nurses’ and nursing students’ mortality perceptions and end-of-life coping competencies: a decade-long systematic review and meta-analysis
Source: Front Med (Lausanne). 2026 May 26;13:1791470. doi: 10.3389/fmed.2026.1791470 (PMC13246359; doi:10.3389/fmed.2026.1791470)

Fig. S1a


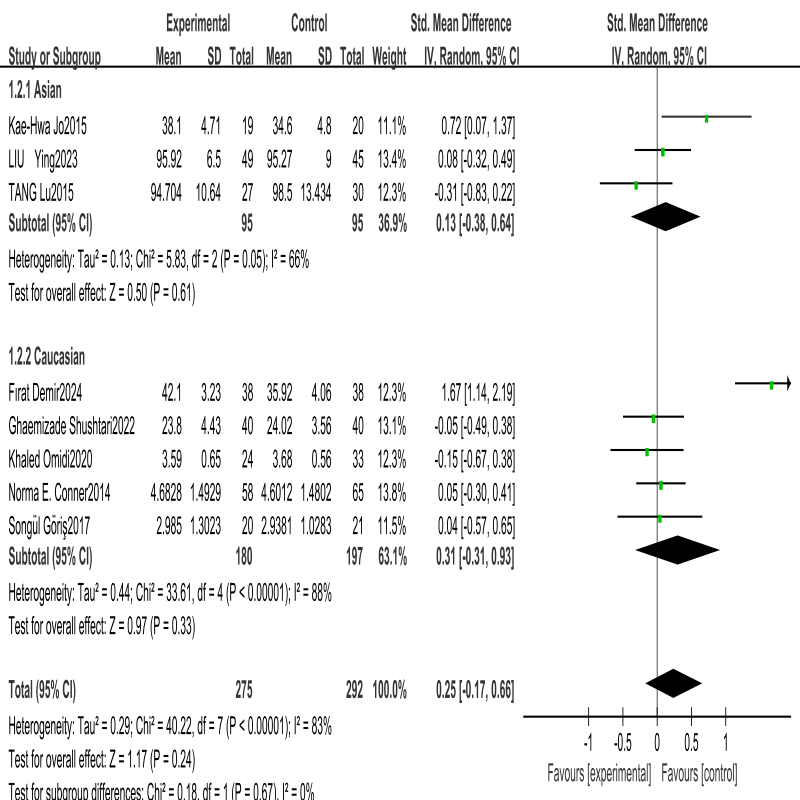


Fig. S1b


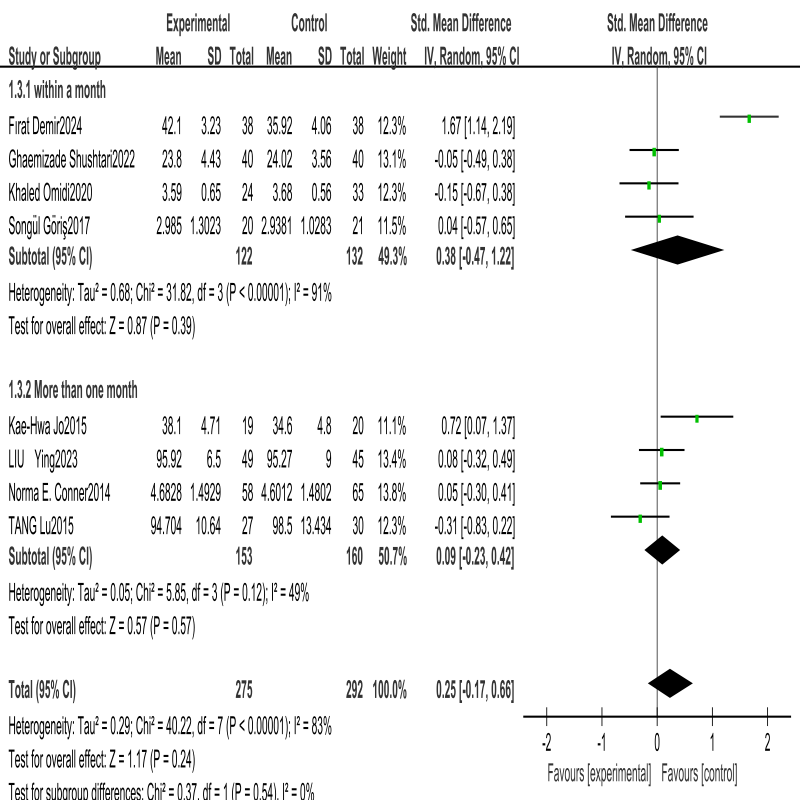


Fig. S1c


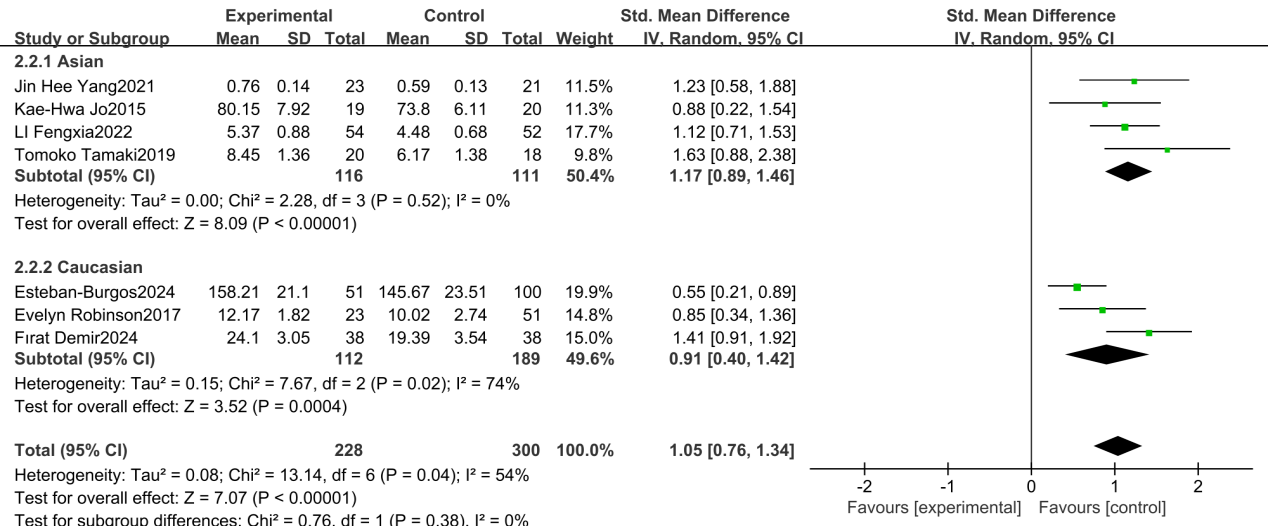


Fig. S1d


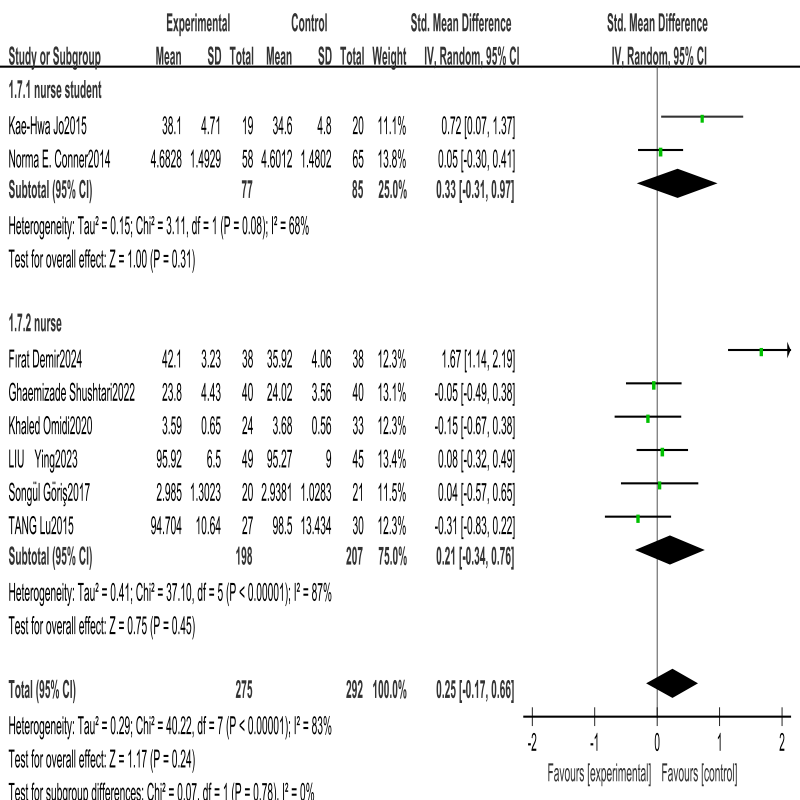


Fig. S2a


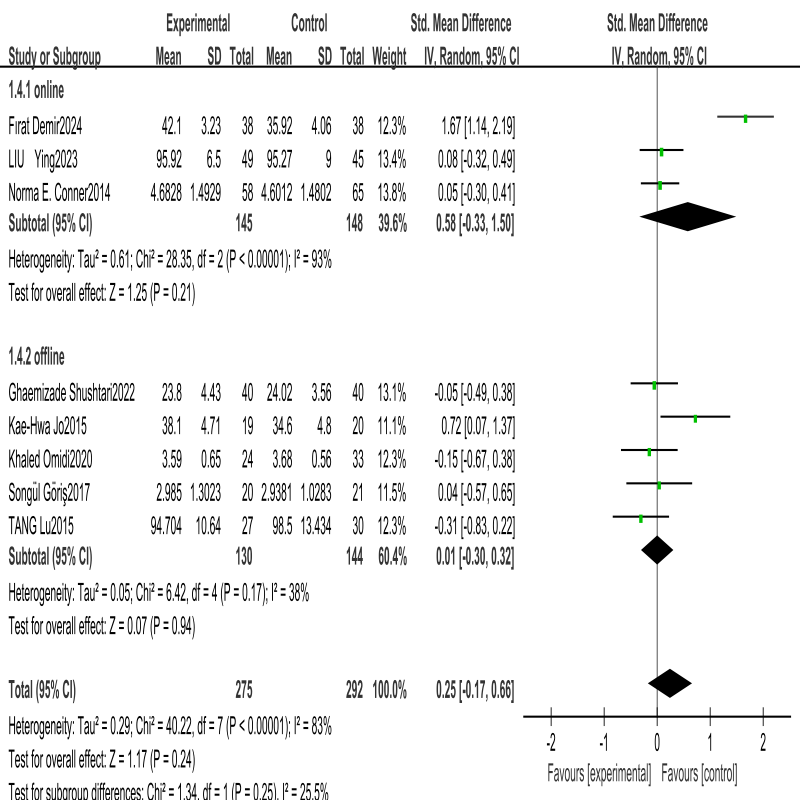


Fig. S2b


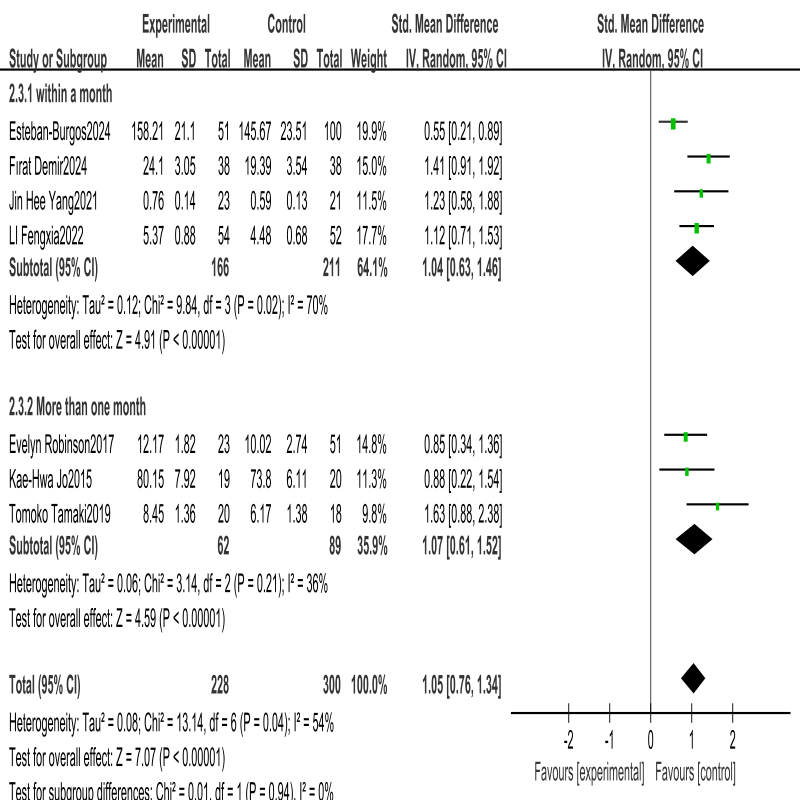


Fig. S2c


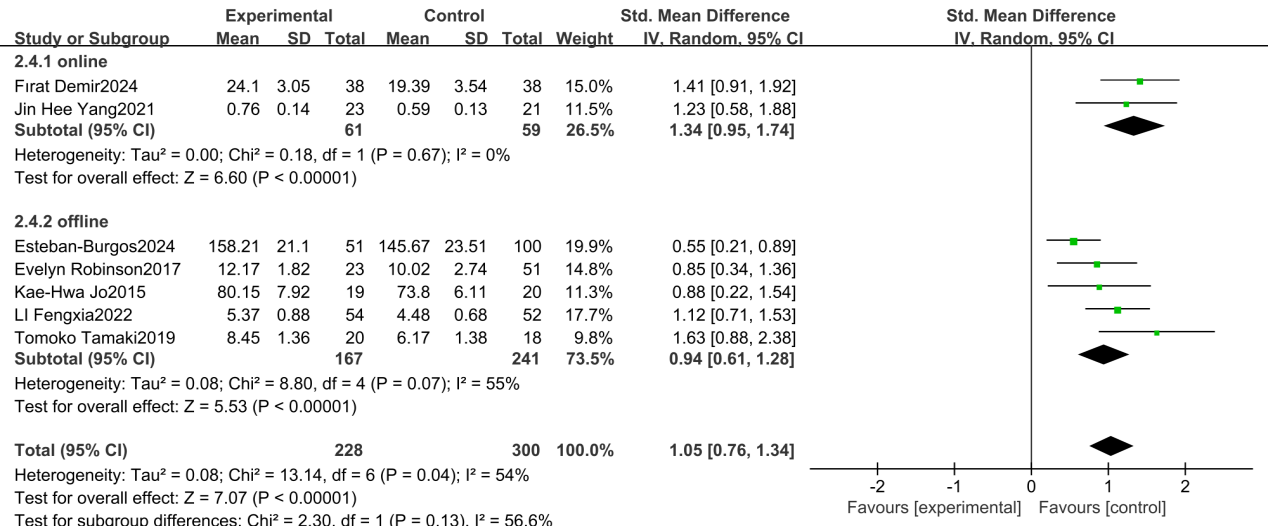


Fig. S2d


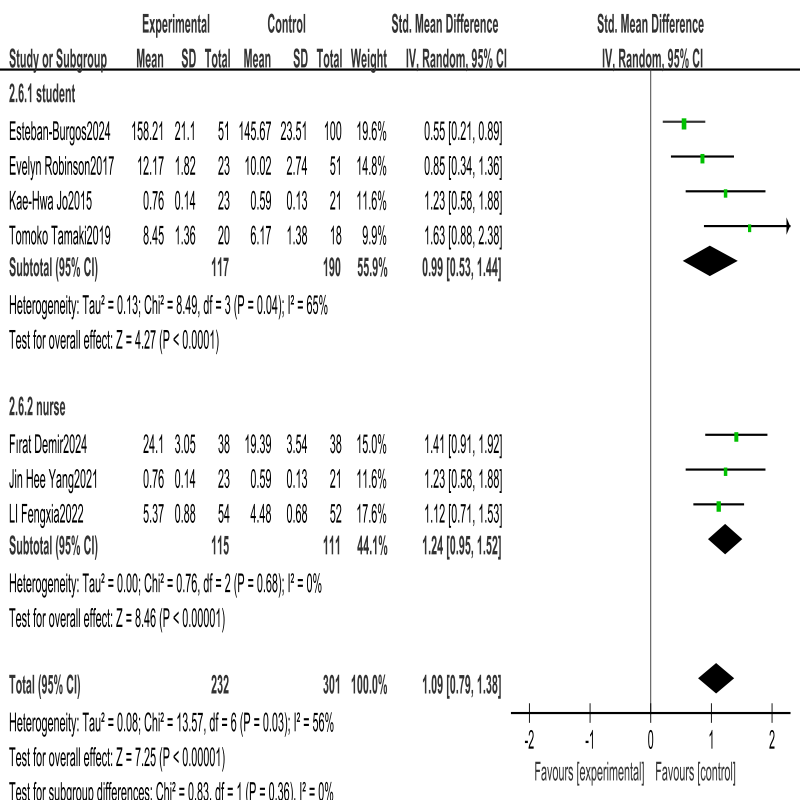


Fig. S2e


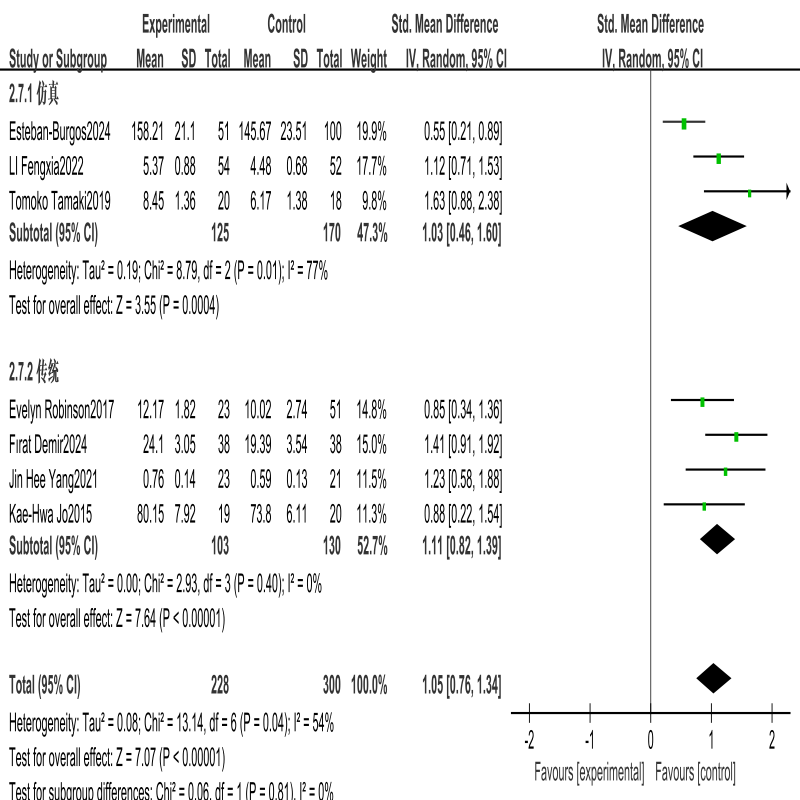


Figure S3


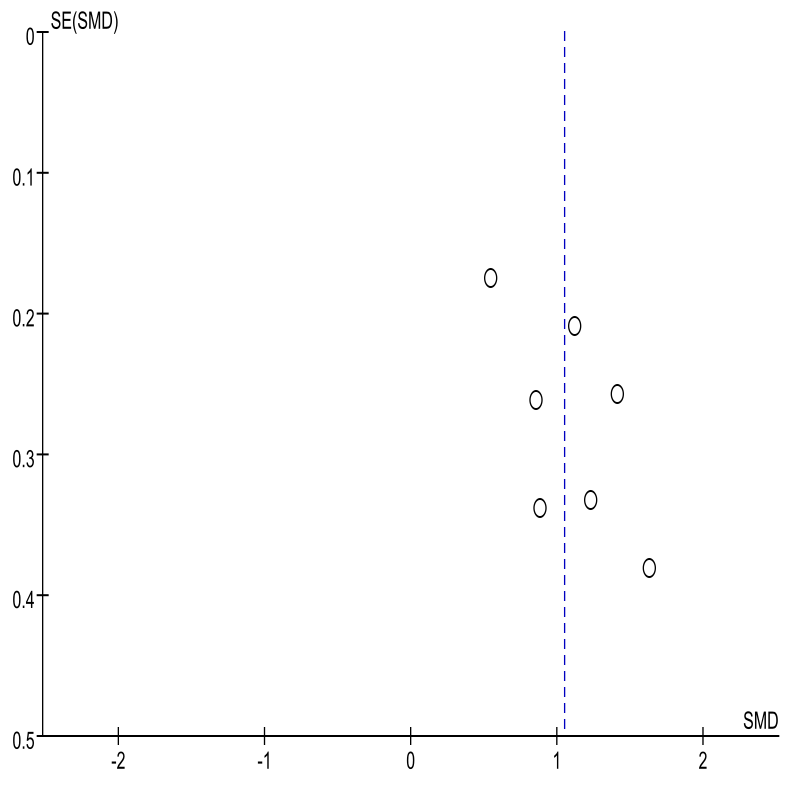


Figure S4


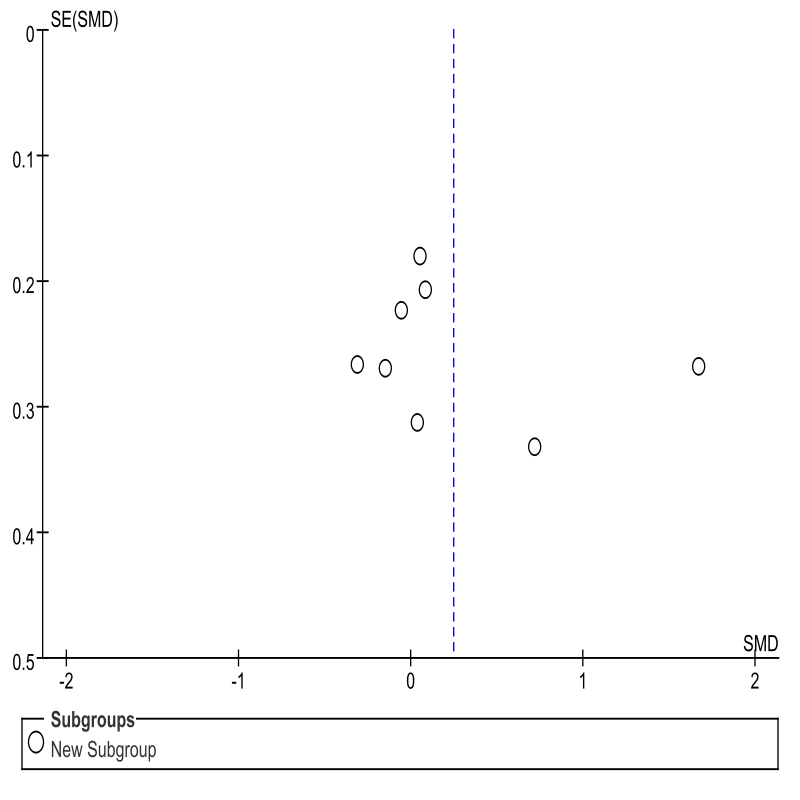

Supplement: Supplementary Figure 1 — (a) Subgroup analysis of attitudes toward death by ethnicity (Asian vs. Caucasian). (b) Subgroup analysis of attitudes toward death by intervention duration (≤1 month vs. >1 month). (c) Subgroup analysis of attitudes toward death by delivery mode (online vs. in-person). (d) Subgroup analysis of attitudes toward death by participant type (nurses vs. nursing students). [file Table_4.docx]
